# Clinical variant interpretation comparing two saturation genome editing-based functional studies for *BRCA2*

**DOI:** 10.3389/fgene.2026.1803717

**Published:** 2026-04-24

**Authors:** Ju Hyeon Shin, Kyung Sun Park, Young-gon Kim, Mi-Ae Jang, Ja-Hyun Jang, Jong-Won Kim

**Affiliations:** 1 Department of Laboratory Medicine and Genetics, Samsung Medical Center, Sungkyunkwan University School of Medicine, Seoul, Republic of Korea; 2 Department of Laboratory Medicine, Chosun University Hospital, Chosun University College of Medicine, Gwangju, Republic of Korea; 3 Department of Laboratory Medicine, Kyung Hee University College of Medicine, Kyung Hee University Hospital, Kyung Hee University Medical Center, Seoul, Republic of Korea

**Keywords:** *BRCA2*, functional evidence, MAVE, saturation genome editing, variant interpretation

## Abstract

**Background:**

Two saturation genome editing (SGE) studies for *BRCA2* using haploid human HAP1 cells and mouse embryonic stem cells, respectively, demonstrated contradictory functional results in 16.9% (1,052/6,208) of the variants. We performed clinical variant interpretation and tried to address the discordance by comparing two studies combined with 24 years of clinicopathological data collected at a single institution.

**Methods:**

Retrospectively, we collected data from patients with *BRCA2* variants evaluated in the SGE studies. The variants were reassessed according to the ClinGen *BRCA1/2* guidelines and/or multifactorial likelihood analysis. For variants with concordant SGE functional results, either PS3 or BS3 was assigned. Major error rates were compared for variants with discordant results.

**Results:**

Among the 88 variants from 526 patients, 13, including three potentially hypomorphic variants, showed discordant results. Major error rates were lower for HAP1-SGE dataset, but without statistical significance. Among the 75 variants with concordant results, 28 and 47 were assigned PS3 and BS3, respectively. Consequently, 93.1% (27/29) of the variants of uncertain significance were reclassified as likely pathogenic (n = 3) or likely benign (n = 24).

**Conclusion:**

Concordant SGE results are clinically useful for variant reclassification. When discordant results are present, functional evidence should not be assigned, but HAP1-SGE dataset is suggested to be more consistent with patient-specific data. Further segregation analysis and long-term follow-up are needed to resolve discordant cases.

## 1 Introduction

Hereditary breast and ovarian cancer syndrome (MIM:604370, MIM:612555) caused by germline pathogenic variants (PVs) of *BRCA1* and *BRCA2* genes (*BRCA1/2*) is associated with increased risk of breast cancer, ovarian cancer, prostate cancer, and pancreatic cancer (Petrucelli et al., 1998). Identification of *BRCA1/2* PV is essential to provide proper management and genetic counseling for patients and family members; however, a vast number of variants remain variants of uncertain significance (VUSs) and provide ambiguity to both clinicians and patients (Dace and Findlay, 2022). Vigorous efforts have been made to reduce VUSs and clarify their clinical effect. For instance, multiplexed assays of variant effect (MAVEs) have enabled high-throughput functional characterization and substantial reduction of VUS (Fayer et al., 2021; Dace and Findlay, 2022). Saturation genome editing (SGE)-based MAVE, using the CRISPR-Cas9 method, covers almost all possible single-nucleotide variants (SNVs) in several target genes, including *BRCA1*, *RAD51C*, *BAP1*, *VHL*, and *TP53* (Findlay et al., 2018; Buckley et al., 2024; Olvera-León et al., 2024; Waters et al., 2024; Funk et al., 2025). However, several challenges and debates remain, including standards for validation and application, acceptable model organisms, and interpretation of contradictory results between different MAVEs (Dace and Findlay, 2022; Allen et al., 2024).

*BRCA2* includes clinically important functional domains that are defined as PALB2 interacting domain (amino acid 10–40) and C-terminal DNA-binding (CTDB) domain (amino acid 2481–3186) where missense PVs are predominantly distributed (ENIGMA *BRCA1*/*2* VCEP, 2025b). Functional evidence, i.e., PS3 or BS3, was not widely applicable for *BRCA2* variants because Clinical Genome Resource (ClinGen) ENIGMA *BRCA1/2* variant curation expert panel (VCEP) calibrated functional evidence for only 531 *BRCA2* variants in contrast to 4,200 *BRCA1* variants which were mostly attributed to the *BRCA1* SGE study (Findlay et al., 2018; ENIGMA *BRCA1/2* VCEP, 2025a; ENIGMA *BRCA1/2* VCEP, 2025b). In January 2025, two SGE studies covering *BRCA2* CTDB domain were published by Huang et al. and Sahu et al., and a total of 6,208 variants intersected the functional data from both studies (Huang et al., 2025; Sahu et al., 2025). Although Huang et al. and Sahu et al. demonstrated high-throughput functional data by using SGE, their methodology was a little different in detail. For example, Huang et al. used haploid human HAP1 cell (HAP1-SGE), whereas Sahu et al. used mouse embryonic stem cell (mES-SGE). Consequently, the functional results of these two studies were contradictory for 1,052 (16.9%) variants.

Here, we clinically interpreted *BRCA2* variants by comparing two SGE functional studies combined with clinicopathological information. Despite single-institution data, 14,821 patients underwent *BRCA1/2* sequencing testing at the Samsung Medical Center (SMC) for 24 years. Using these data, we analyzed and addressed the contradictory SGE results. In addition, we examined the clinical utility of the MAVE for *BRCA2* variant classification.

## 2 Materials and methods

### 2.1 Data collection

This study initially included 14,821 patients who underwent *BRCA1/2* sequencing at the SMC between September 2001 and December 2024. The samples were either obtained from patients visiting the SMC or requested from external hospitals. Then, we selected patients who had *BRCA2* variants that were evaluated in the two SGE studies (Huang et al., 2025; Sahu et al., 2025). Electronic medical records were retrospectively reviewed to collect the personal medical history, clinicopathological characteristics of breast and/or ovarian cancer, and family history of cancer. Specifically, age at diagnosis, cancer type and site, histology, histologic grade (Bloom–Richardson grade), estrogen-receptor status, progesterone-receptor status, human epidermal growth factor receptor 2 status, and Ki-67 grade were obtained.

### 2.2 Direct sequencing for *BRCA1/2* genes

Direct sequencing was performed at the SMC as previously described (Bang et al., 2022). Briefly, before September 2016, *BRCA1/2* Sanger sequencing was performed using ABI Prism 3130xl or 3730xl DNA Analyzer (Applied Biosystems, Foster City, CA, United States). Genomic DNA was extracted and purified from peripheral blood leukocytes. All coding exons and their flanking introns were amplified and sequenced. The sequences were compared with reference sequences using Sequencher software (Gene Codes Co., Ann Arbor, MI, United States). Between September 2016 and July 2023, *BRCA1/2* next-generation sequencing was performed using the Ion S5 XL Sequencer with the Oncomine BRCA assay (Thermo Fisher Scientific, Waltham, MA, United States) and from August 2023, it was performed using the Genexus Integrated Sequencer with the Oncomine BRCA assay (Thermo Fisher Scientific) following the manufactures’ instructions. The sequences were compared to the *BRCA1* (NM_007294.4) and *BRCA2* (NM_000059.4) reference sequences. Variants were described according to the recommendations of the Human Genome Variation Society.

### 2.3 Variant assessment

Previously reported variants were reassessed according to the American College of Medical Genetics and Genomics/Association for Molecular Pathology guidelines and ClinGen *BRCA1*/*2* guidelines (Richards et al., 2015; ENIGMA *BRCA1/2* VCEP, 2025a; ENIGMA *BRCA1/2* VCEP, 2025b). When both pathogenic and benign evidence was present, the variant was classified using a point-based approach (Tavtigian et al., 2020). Pathogenic evidence was converted to points according to its strength: supporting, moderate, strong, and very strong evidence were awarded 1, 2, 4, and 8 points, respectively. Benign evidence was converted in the same manner: supporting, moderate, strong, and very strong evidence were awarded −1, −2, −4, and −8 points, respectively. Final classification was determined based on the aggregated points: PV ≥ 10; likely PV (LPV) 6–9; VUS -1 to 5; likely benign variant (LBV) −6 to −2; benign variant (BV) ≤ −7 (ENIGMA *BRCA1/2* VCEP, 2025a; ENIGMA *BRCA1/2* VCEP, 2025b).

For additional assignment of functional evidence, we compared HAP1-SGE and mES-SGE functional datasets (Huang et al., 2025; Sahu et al., 2025). Both studies classified the functional results as seven categories including pathogenic strong, pathogenic moderate, pathogenic supporting, uncertain, benign supporting, benign moderate, and benign strong. When HAP1-SGE and mES-SGE functional results were concordant for a variant, that is, both pathogenic and benign, we assigned either PS3 or BS3. We compared the reclassification rates with and without concordant functional evidence, except for variants that had been reviewed by an expert panel in ClinVar (accessed February 28, 2025). In contrast, when HAP1-SGE and mES-SGE functional results were discordant, no functional evidence was assigned. Instead, major error, minor error, and concordance rates between variant classification and SGE functional results were compared. A major error was defined either as a pathogenic functional result for BV/LBV or as a benign functional result for PV/LPV. A minor error was defined either as an uncertain functional result for PV/LPV and BV/LBV or as a pathogenic or benign functional result for VUS. The Bhapkar test was used to compare major error rates between the two sets of SGE data. Statistical analysis was performed using R software (version 4.4.2), and *P* < 0.05 was considered statistically significant.

Multifactorial likelihood analysis was additionally performed for variants with discordant SGE functional results based on the previous KONCORD study (Park et al., 2016; Park et al., 2021). The multifactorial probability of pathogenicity was calculated using prior probability and combined likelihood ratio (LR) based on Bayes theorem (Lindor et al., 2012). We applied prior probability using Align-GVGD grades (Lindor et al., 2012). For synonymous, intronic, and nonsense variants with no Aling-GVGD grades, applicable prior probability was obtained from Huntsman Cancer Institute databases (https://hci-priors.hci.utah.edu/PRIORS/BRCA/indexBRCA2.php (accessed July 21, 2025)) (Vallée et al., 2016). Combined LR was calculated from LRs for personal/family cancer history, pathologic profiles, and co-occurrence with pathogenic variants between benign and pathogenic groups collected in the KONCORD study (Park et al., 2021). The benign group was defined as individuals with only BVs/LBVs in *BRCA1/2*, whereas the pathogenic group was defined as individuals with PVs/LPVs in *BRCA1* or *BRCA2*. The International Agency for Research on Cancer (IARC) classification was determined as follows: posterior probability of >0.99 as class 5 (pathogenic); 0.95–0.99 as class 4 (likely pathogenic); 0.05–0.949 as class 3 (uncertain); 0.001–0.049 as class 2 (likely neutral); and <0.001 as class 1 (neutral). Because multifactorial likelihood analysis is highly affected by prior probability especially for a rare variant with insufficient number of patients, variants with a combined LR between 0.5 and 2 were excluded for comparing major error, minor error, and concordance rates according to the previous studies (Vallée et al., 2016; Park et al., 2021).

## 3 Results

### 3.1 Overall results

**FIGURE 1:**
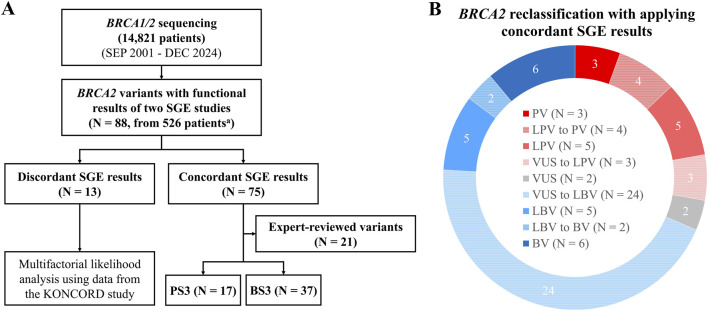
Flowchart and pie chart showing the collection of *BRCA2* variants and application of SGE functional data in this study. **(A)** A total of 88 variants had SGE functional results from two studies (Huang et al., 2025; Sahu et al., 2025). Among them, 13 variants showed discordant results. For these variants, additional multifactorial likelihood analysis was performed using data from the KONCORD study (Park et al., 2021). Either PS3 or BS3 was assigned to 75 variants with concordant results, 21 of which had been reviewed by expert panel. Among the remaining 54 variants, 17 variants were assigned PS3, and 37 variants were assigned BS3. **(B)** Assigning PS3 or BS3, 33 variants were reclassified as follows: from LPV to PV (n = 4), from VUS to LPV (n = 3), from VUS to LBV (n = 24), from LBV to BV (n = 2) ^a^All patients except for eight had only one SGE variant. The remaining eight patients had two SGE variants simultaneously. Abbreviations: BV, benign variant; LBV, likely benign variant; LPV, likely pathogenic variant; PV, pathogenic variant; SGE, saturation genome editing; VUS, variant of uncertain significance.

A schematic flowchart shows the collection of *BRCA2* variants and the application of SGE functional data in this study (Figure 1A). A total of 88 variants from 526 patients had functional results from HAP1-SGE and mES-SGE studies (Huang et al., 2025; Sahu et al., 2025). Of 88 variants, 13 variants had discordant SGE results. For these variants, additional multifactorial likelihood analysis was performed using data from the KONCORD study (Park et al., 2021). Either PS3 or BS3 was assigned to 75 variants with concordant SGE results, 21 of which had been reviewed by expert panel and were excluded from variant reassessment in this study. Among the remaining 54 variants, 17 variants were assigned PS3, and 37 variants were assigned BS3. All patients except for eight had only one SGE variant. The remaining eight patients had two SGE variants as follows. Five patients with c.7469T>C p.(Ile2490Thr) also carried c.7480C>T p.(Arg2494*) (n = 2), c.7661G>T p.(Ser2554Ile) (n = 1), c.7870T>A p.(Tyr2624Asn) (n = 1), or c.8499G>A p.(Lys2833=) (n = 1), respectively. Another two patients with c.8851G>A p.(Ala2951Thr) carried c.7480C>T p.(Arg2494*) and the other one with c.7814G>A p.(Cys2605Tyr) had c.8165C>T p.(Thr2722Ile). Among them, c.7661G>T p.(Ser2554Ile) and c.8499G>A p.(Lys2833 =) were variants with discordant SGE results.

### 3.2 Variants with discordant SGE functional results

Thirteen variants with discordant results were obtained from 56 patients (Table 1). These variants were initially classified as two PVs, seven VUSs, and four LBVs, according to the ClinGen *BRCA1*/*2* guidelines. Compared to the initial classification, one PV, one VUS, and three LBVs showed consistent functional results from the HAP1-SGE dataset, whereas one PV, three VUSs, and one LBV showed consistent functional results from the mES-SGE dataset. The major error, minor error, and concordance rates for the HAP1-SGE functional results were 15.4%, 46.2%, and 38.5%, respectively, and those for the mES-SGE functional results were 23.1%, 38.5%, and 38.5%, respectively. The major error rates were not significantly different (*P* = 1.0).

**TABLE 1:** *BRCA2* variants with discordant functional results between the HAP1-SGE and mES-SGE studies.

Nucleotide change^a^	Amino acid change	N of patients	Evaluation without applying SGE results			SGE functional result		Multifactorial likelihood analysis			ClinVar
			ACMG/AMP criteria	Points	Classification	Points (HAP1-SGE)	Points (mES-SGE)	Combined LR	Posterior probability of pathogenicity	IARC class	
c.7436–4A>G	p.?	2	BP4, BP7_S (RNA)	−5	LBV	−4	2	1.493	0.435	NA^b^	VUS; LBV; BV
c.7481G>T	p.(Arg2494Leu)	1	PM2_P, BP4	0	VUS	−4	4	0.684	0.745	NA^b^	VUS
c.7661G>T	p.(Ser2554Ile)	1	PM2_P	1	VUS	−4	2	1.030	0.296	NA^b^	NA
c.7691C>G	p.(Thr2564Ser)	27	BS1_P, BS2_P, BP4	−3	LBV	−4	2	0.000	0.000	1	VUS
c.7768T>C	p.(Ser2590Pro)	1	PM2_P, BP4	0	VUS	0	−4	0.253	0.008	2	VUS
c.8156T>C	p.(Ile2719Thr)	1	BP4	−1	VUS	−4	0	0.684	0.218	NA^b^	VUS
c.8342A>G	p.(Asn2781Ser)	5	PM2_P, BP4	0	VUS	4	0	0.512	0.499	NA^b^	LPV; VUS
c.8499G>A	p.(Lys2833=)	1	BS1, BP4, BP7	−6	LBV	−4	0	0.253	0.005	2	LBV
c.8944A>C	p.(Lys2982Gln)	5	BS1_P, BP4	−2	LBV	4	−4	0.024	0.001	1	VUS; LBV
c.8991T>G	p.(Tyr2997*)	9	PVS1, PM2_P, PM5_S	13	PV	4	−4	29.348	0.999	5	PV
c.9117+1G>A	p.?	1	PVS1 (RNA), PS1_P, PM2_P	10	PV	−2	4	NA^c^	NA^c^	NA^c^	PV
c.9232G>T	p.(Val3078Phe)	1	PM2_P	1	VUS	4	0	0.778	0.023	NA^b^	VUS
c.9533A>C	p.(Asn3178Thr)	1	PM2_P, BP4	0	VUS	2	−4	1.030	0.031	NA^b^	NA

^a^ Reference sequence: NM_000059.4.

^b^ Variants with a combined LR, between 0.5 and 2 were not assigned IARC, class.

^c^ The c.9117+1G>A variant was excluded from multifactorial likelihood analysis because the patient’s clinical information was not sufficiently provided by an external hospital.

Abbreviations: ACMG/AMP, American College of Medical Genetics and Genomics/Association for Molecular Pathology; BV, benign variant; HAP1-SGE, haploid human HAP1 cell-based saturation genome editing; IARC, international agency for research on cancer; LBV, likely benign variant; LPV, likely pathogenic variant; LR, likelihood ratio; mES-SGE, mouse embryonic stem cell-based saturation genome editing; NA, not available; PV, pathogenic variant; SGE, saturation genome editing; VUS, variant of uncertain significance.

The SGE functional results were additionally compared to the IARC class based on multifactorial likelihood analysis. The c.9117+ 1G>A was excluded for analysis because the patient’s clinical information was not sufficiently provided by an external hospital. Seven variants with a combined LR between 0.5 and 2 were not assigned IARC class. Finally, five variants could be classified according to IARC class. IARC class of four (80.0%) variants were consistent with the initial classification based on ClinGen *BRCA1/2* guidelines, whereas one initial VUS was reclassified as IARC class 2. Compared to the IARC class, the major error, minor error, and concordance rates for the HAP1-SGE functional results were 20.0%, 20.0%, and 60.0%, respectively, and those for the mES-SGE functional results were 40.0%, 20.0%, and 40.0%, respectively (*P* = 0.84).

Among four variants with uncertain functional results from the mES-SGE dataset, except for a synonymous variant c.8499G>A p.(Lys2833=), three variants were presented as potentially hypomorphic variants that showed sensitivity to DNA-damaging agents (Sahu et al., 2025). One variant (c.8156T>C p.(Ile2719Thr)) showed benign functional result and two variants (c.8342A>G p.(Asn2781Ser) and c.9232G>T p.(Val3078Phe)) showed pathogenic functional results from the HAP1-SGE study (Huang et al., 2025). None of the patients had a family history of breast, ovarian, prostate, or pancreatic cancer except for two probands with c.8342A>G p.(Asn2781Ser) (Supplementary Table S1). These two probands had one or two sisters diagnosed with breast cancer, respectively, but *BRCA1/2* sequencing testing of affected family members were not performed.

### 3.3 Variants with concordant SGE functional results

Among the 75 variants with concordant results, 21 expert-reviewed variants showed functional results consistent with their classification (Supplementary Table S2). By assigning PS3 or BS3 to the remaining 54 variants, 33 were reclassified, as shown in Figure 1B. In addition, 93.1% (27/29) of the VUSs were reclassified as LPVs (n = 3) or LBVs (n = 24) (Table 2). All variants, except VUS, showed functional results consistent with their initial classification (Supplementary Table S3).

**TABLE 2:** Reassessment of 29 VUSs by assigning concordant SGE functional evidence.

Final reclassification	Nucleotide change^a^	Amino acid change	N of patients	Evaluation with applying SGE results				ClinVar
				Points (HAP1-SGE)	Points (mES-SGE)	Integrated functional evidence	Final ACMG/AMP criteria	
LPV	c.7805G>A	p.(Arg2602Lys)	1	4	4	PS3	PS1_M, PS3, PP3	VUS
	c.8069T>A	p.(Val2690Asp)	1	4	4	PS3	PS3, PM2_P, PP3	NA
	c.9221T>C	p.(Leu3074Pro)	6	4	4	PS3	PS3, PM2_P, PP1_M, PP3	LPV
VUS	c.7523G>T	p.(Gly2508Val)	1	4	4	PS3	PS3, PM2_P	NA
	c.7871A>G	p.(Tyr2624Cys)	1	4	2	PS3	PS3, PP3	LPV; VUS
LBV	c.7469T>A	p.(Ile2490Lys)	1	−4	−4	BS3	PM2_P, BS3, BP4	NA
	c.7490A>C	p.(Lys2497Thr)	2	−4	−4	BS3	PM2_P, BS3, BP4	VUS
	c.7590A>G	p.(Gln2530=)	1	−4	−4	BS3	PM2_P, BS3, BP4, BP7	LBV
	c.7592T>C	p.(Val2531Ala)	1	−4	−4	BS3	PM2_P, BS3, BP4	NA
	c.7671A>C	p.(Ala2557=)	1	−4	−4	BS3	PM2_P, BS3, BP4, BP7	LBV
	c.7735A>G	p.(Ile2579Val)	1	−4	−4	BS3	PM2_P, BS3, BP4	VUS
	c.7814G>A	p.(Cys2605Tyr)	8	−2	−4	BS3	BS3	VUS
	c.7901T>A	p.(Met2634Lys)	3	−4	−4	BS3	PP3, BS3	VUS; LBV
	c.8047G>C	p.(Ala2683Pro)	1	−4	−4	BS3	PM2_P, PP3, BS3	NA
	c.8215G>C	p.(Val2739Leu)	1	−4	−4	BS3	BS3, BP4	VUS; LBV
	c.8428A>C	p.(Ser2810Arg)	1	−4	−4	BS3	PM2_P, BS3, BP4	VUS
	c.8488–3C>A	p.?	1	−4	−4	BS3	PM2_P, PP3, BS3	VUS
	c.8587G>A	p.(Glu2863Lys)	1	−4	−4	BS3	PM2_P, PP3, BS3	NA
	c.8744C>T	p.(Ala2915Val)	1	−4	−4	BS3	PM2_P, BS3, BP4	VUS
	c.8914T>C	p.(Leu2972=)	1	−4	−4	BS3	PM2_P, BS3, BP4, BP7	LBV
	c.8968T>C	p.(Trp2990Arg)	1	−4	−4	BS3	PM2_P, BS3	NA
	c.9100C>G	p.(Gln3034Glu)	1	−4	−4	BS3	PM2_P, BS3, BP4	VUS
​	c.9111A>G	p.(Gln3037=)	1	−4	−4	BS3	PM2_P, BS3, BP4, BP7	LBV
	c.9161C>G	p.(Pro3054Arg)	1	−4	−4	BS3	PM2_P, BS3, BP4	VUS
	c.9166C>T	p.(His3056Tyr)	1	−4	−4	BS3	BS3, BP4	VUS
	c.9309A>G	p.(Ile3103Met)	1	−4	−4	BS3	PM2_P, BS3, BP4	VUS
	c.9403C>G	p.(Leu3135Val)	3	−4	−4	BS3	PM2_P, BS3, BP4	NA
	c.9485T>G	p.(Met3162Arg)	1	−4	−4	BS3	PM2_P, BS3	NA
	c.9544C>T	p.(His3182Tyr)	2	−4	−4	BS3	PM2_P, BS3, BP4	VUS

^a^ Reference sequence: NM_000059.4.

Abbreviations: ACMG/AMP, American College of Medical Genetics and Genomics/Association for Molecular Pathology; HAP1-SGE, haploid human HAP1 cell-based saturation genome editing; LBV, likely benign variant; LPV, likely pathogenic variant; mES-SGE, mouse embryonic stem cell-based saturation genome editing; NA, not available; SGE, saturation genome editing; VUS, variant of uncertain significance.

With assignment of PS3, three VUSs (c.7805G>A p.(Arg2602Lys), c.8069T>A p.(Val2690Asp), and c.9221T>C p.(Leu3074Pro)) were reclassified as LPVs (Table 2). Two of three reclassified LPVs met other pathogenic evidence in addition to PM2_supporting and/or PP3. The c.7805G>A p.(Arg2602Lys), located in the last nucleotide of exon 16, was predicted to cause the skipping of exon 16 and deletion of the last 100 nucleotides using SpliceAI (Jaganathan et al., 2019). PS1_moderate was assigned based on a previously reported LPV, c.7805G>C p.(Arg2602Thr), with the same predicted and proven impact on splicing using a minigene assay (Fraile-Bethencourt et al., 2018). The c.9221T>C p.(Leu3074Pro) was detected in six patients, three of whom were family members diagnosed with breast cancer, which enabled PP1_moderate assignment (Supplementary Figure S1). The other reclassified LPV, c.8069T>A p.(Val2690Asp) was detected in a patient diagnosed with unilateral breast cancer at the age of 35 years and having two family members with breast cancer (sister and paternal aunt).

With assignment of BS3, 24 VUSs were reclassified as LBVs (Table 2). Among them, 21 variants met both pathogenic and benign evidence; however, all variants were finally reclassified as LBVs based on a point-based system. The assigned pathogenic evidence was PM2_supporting for 17 variants, PP3 for one variant, and PM2_supporting and PP3 for three variants. Three variants with PM2_supporting, PP3, and BS3 evidence were c.8047G>C p.(Ala2683Pro), c.8488–3C>A, and c.8587G>A p.(Glu2863Lys). A patient with c.8047G>C p.(Ala2683Pro) who had been diagnosed with ovarian cancer underwent whole genome sequencing and was found to have a heterozygous *RAD51* PV (NM_002878.4:c.270_271dup p.(Lys91Ilefs*13)).

## 4 Discussion

*BRCA2* was the first gene to be evaluated by two different study groups using SGE-based MAVE. However, 16.9% of the variants with discordant results presented an unresolved issue. To determine the functional dataset more consistent with the patient-specific data, we performed a clinical application of different SGE studies on *BRCA2* by combining clinicopathological data. As expected, in line with previous SGE reports for *BRCA1* (Fayer et al., 2021; Dace and Findlay, 2022), concordant SGE results enabled substantial reclassification of VUS. However, 13 variants showed discordant results and could not be assigned consistent functional evidence. This indicates that interpreting variants based solely on either HAP1-SGE or mES-SGE dataset may be inaccurate. Nevertheless, the major error rate was lower for the HAP1-SGE dataset than for the mES-SGE dataset despite no statistical significance and insufficient number for generalization to the other 1,039 variants with discordant results. This implies that acceptable model organisms should be considered for validation of SGE-based MAVE. As more SGE studies are performed, more contradictory results will be produced, which will confuse clinicians when interpreting variants with discordant results (Dace and Findlay, 2022). Standards and guidelines for the validation and application of MAVE are required to minimize future uncertainty.

The discordance between two SGE studies can be attributed to their different methodology (Huang et al., 2025; Sahu et al., 2025). First, the HAP1-SGE study adjusted the VarCall model to determine pathogenicity, assuming that silent variants with no aberrant splice effects are benign and nonsense variants are pathogenic (Huang et al., 2025). The mES-SGE study trained and calibrated a statistical model using 12 SNVs classified according to a multifactorial likelihood analysis (Sahu et al., 2025). Consequently, c.8499G>A p.(Lys2833=) and c.8991T>G p.(Tyr2997*) showed benign and pathogenic functional results, respectively, in the HAP1-SGE study, whereas they showed uncertain and benign functional results, respectively, in the mES-SGE study. Meanwhile, the canonical splice site PV c.9117+1G>A had benign and pathogenic functional results in the HAP1-SGE and mES-SGE studies, respectively. In addition, among 21 nonsense variants and 32 canonical splice site variants with discordant SGE results, 100% and 62.5% showed pathogenic functional results in the HAP1-SGE study, respectively (data not shown).

Second, two studies used different cell types. Huang et al. used haploid human HAP1 cells because *BRCA2* is involved in homology-directed DNA repair (HDR) pathway and one of the essential genes for HAP1 cells (Blomen et al., 2015; Dace and Findlay, 2022; Huang et al., 2025). Other SGE studies also have used HAP1 cells to evaluate *BRCA1* and *RAD51C* which are associated with the HDR pathway and are essential for HAP1 cells like *BRCA2* (Findlay et al., 2018; Olvera-León et al., 2024). In addition, SGE studies for *DDX3X*, *BAP1*, and *VHL* used HAP1 cells (Radford et al., 2023; Buckley et al., 2024; Waters et al., 2024), while SGE studies for *CARD11* and *TP53* used non-HAP1 human cells (Meitlis et al., 2020; Funk et al., 2025). On the other hand, Sahu et al. used mouse embryonic stem cells (Sahu et al., 2025). Relevant experts disagree with restricting the acceptable model organisms for MAVE if the validation data are robust (Allen et al., 2024). However, thorough functional assay can be limited when variants are tested outside their endogenous genomic context, depending on the underlying mechanism of the disease (Dace and Findlay, 2022; Yang et al., 2024). Recently, SGE-based MAVE using human induced pluripotent stem cells demonstrated functional assessment of *MYBPC3* and *POLG* variants in the disease-relevant cell types and underscored the importance of genetic contexts (Fayer et al., 2025). Using human cells might mitigate the confounding effect of endogenous and intracellular elements on human variant interpretation.

Three variants with discordant functional results were suggested as hypomorphic variants by Sahu et al. because cells with these variants typically proliferated but exhibited sensitivity to cisplatin and olaparib (Sahu et al., 2025). These variants were classified as VUS and were not available for the IARC class in this study; however, they might be reclassified as PV/LPVs with a lower cancer risk in the future. *BRCA1* c.5096G>A p.(Arg1699Gln), which was initially classified as IARC class 3, was later found to exhibit an intermediate risk of breast and ovarian cancers based on segregation analysis of 129 families (Moghadasi et al., 2018). Moreover, based on case–control and segregation studies, *BRCA2* c.9104A>C p.(Tyr3035Ser) was considered to confer an intermediate risk and demonstrated vulnerability to DNA-damaging agents in the mES-SGE study (Shimelis et al., 2017; Sahu et al., 2025). Although hypormophic variants are associated with a lower cancer risk, patients with these variants may benefit from PARP inhibitors (Sahu et al., 2025). Considering that two potentially hypomorphic variants (c.8342A>G p.(Asn2781Ser) and c.9232G>T p.(Val3078Phe)) showed pathogenic functional results from the HAP1-SGE study, these variants were supposed to have pathogenic characteristics. If potentially hypomorphic variants in the mES-SGE study show pathogenic functional results from the HAP1-SGE study, further evaluation of family members and long-term follow-up are needed to elucidate the pathogenicity.

In this study, variants with concordant results were assigned functional evidence at the strong level such as PS3 or BS3 because at least one SGE functional result belonged to the strong level category. However, downgrading evidence, such as PS3_supporting or BS3_supporting, should be considered when integrating SGE functional results at the supporting level category. It is debatable to classify rare variants as PV/LPVs based solely on MAVE functional evidence combined with *in silico* and population frequency data (Allen et al., 2024). Because it was considered that variant assessment without patient-specific data might lead to misclassification, it was proposed that the final classification should be provided by a clinician who combines patient-specific data (Allen et al., 2024). In this study, SGE functional evidence was in line with pre-existing evidence for reclassified LPVs (Table 2). In contrast, 21 reclassified LBVs met additional PM2_supporting and/or PP3. Among three reclassified LBVs with contradictory evidence (PM2_supporting, PP3, and BS3), c.8047G>C p.(Ala2683Pro) was suggested to have benign characteristics because of its co-occurrence with a heterozygous *RAD51* PV. Another variant c.8488–3C>A is predicted to cause exon 20 skipping with delta scores of 0.28 using SpliceAI (Jaganathan et al., 2019). However, considering a low delta score and concordant benign SGE functional results, we speculated that c.8488–3C>A would result in small proportion of aberrant transcripts to the wild type. Unfortunately, its splicing effect was not confirmed by RNA analysis because of follow-up loss of the proband.

ClinGen *BRCA1/2* guidelines suggested that PS3/BS3 for intronic and synonymous variants should be considered only from assays that measure effect via both mRNA and protein (ENIGMA *BRCA1*/*2* VCEP, 2025a; ENIGMA *BRCA1*/*2* VCEP, 2025b). According to a previous *BRCA1* SGE study, by measuring cell fitness, the effects of variants can be demonstrated integrating multiple layers of gene function such as splicing (Findlay et al., 2018). In that study, function scores based on genomic DNA were compared with RNA scores based on cDNA. Exonic variants inducing aberrant splicing and 12 synonymous variants that were classified as non-functional based on function scores were also shown to reduce mRNA expression. By this observation, Findlay et al. assumed that non-functional variants with low mRNA levels would affect RNA splicing (Findlay et al., 2018). Likewise, although *BRCA2* SGE studies did not conduct direct RNA splicing analysis, several variants predicted to affect splicing were shown to be functionally pathogenic (Huang et al., 2025; Sahu et al., 2025). Collectively, we considered that SGE functional results were reflected via multiple genetic effects on mRNA and protein. Therefore, we assigned PS3/BS3 to intronic and synonymous variants in line with application of *BRCA1* SGE dataset (ENIGMA *BRCA1/2* VCEP, 2025a; Findlay et al., 2018).

This study had some limitations. Owing to the retrospective nature of the study, a thorough evaluation of co-segregation and RNA splicing could not be achieved. Second, we did not assign PP4/BP5 evidence to minimize the bias because multifactorial likelihood analysis was performed using the relatively small number (5433 participants) of data in the KONCORD study compared to the large-scale case-control study including more than 400,000 participants (Park et al., 2021; Zanti et al., 2025). Third, a multiplex ligation-dependent probe amplification assay was not routinely performed for patients at the SMC because of insurance and financial constraints. However, considering the low prevalence of large genomic rearrangements in Korea, which has been reported to be approximately 1% (Park et al., 2017), the confounding effect was not assumed to be significant.

In conclusion, the application of concordant functional results between the HAP1-SGE and mES-SGE studies is clinically useful for variant reclassification. When discordant results are present, functional evidence should not be assigned, but HAP1-SGE dataset is suggested to be more consistent with patient-specific data. Further segregation analysis and long-term follow-up are needed to resolve discordant cases.

## Data Availability

The original contributions presented in the study are included in the article/Supplementary Material, further inquiries can be directed to the corresponding author.
