# Correction: Clinical variant interpretation comparing two saturation genome editing-based functional studies for *BRCA2*

**DOI:** 10.3389/fgene.2026.1895381

**Published:** 2026-06-12

**Authors:** Ju Hyeon Shin, Kyung Sun Park, Young-gon Kim, Mi-Ae Jang, Ja-Hyun Jang, Jong-Won Kim

**Affiliations:** 1 Department of Laboratory Medicine and Genetics, Samsung Medical Center, Sungkyunkwan University School of Medicine, Seoul, Republic of Korea; 2 Department of Laboratory Medicine, Chosun University Hospital, Chosun University College of Medicine, Gwangju, Republic of Korea; 3 Department of Laboratory Medicine, Kyung Hee University College of Medicine, Kyung Hee University Hospital, Kyung Hee University Medical Center, Seoul, Republic of Korea

**Keywords:** *BRCA2*, functional evidence, MAVE, saturation genome editing, variant interpretation

In the published article, there was a mistake in the **Funding** statement. The funding statement for the National Bio Bigdata Project, funded by four ministries (Ministry of Health and Welfare, Ministry of Science and ICT, Ministry of Trade, Industry and Energy, and Korea Disease Control and Prevention Agency) of Korea was incorrect. The correct grant number is RS-2024-00459500.

The **Funding** statement should read: The author(s) declared that financial support was received for this work and/or its publication. This research was supported by a grant of National Bio Bigdata Project, funded by four ministries (Ministry of Health and Welfare, Ministry of Science and ICT, Ministry of Trade, Industry and Energy, and Korea Disease Control and Prevention Agency) of Korea (grant number: RS-2024-00459500).

The original article has been updated.

