# Supplementary material for: Clinical variant interpretation comparing two saturation genome editing-based functional studies for BRCA2
Source: Front Genet. 2026 Apr 24;17:1803717. doi: 10.3389/fgene.2026.1803717 (PMC13152255; doi:10.3389/fgene.2026.1803717)
Supplement: Supplementary file 2 [file DataSheet1.docx]

Supplementary Material


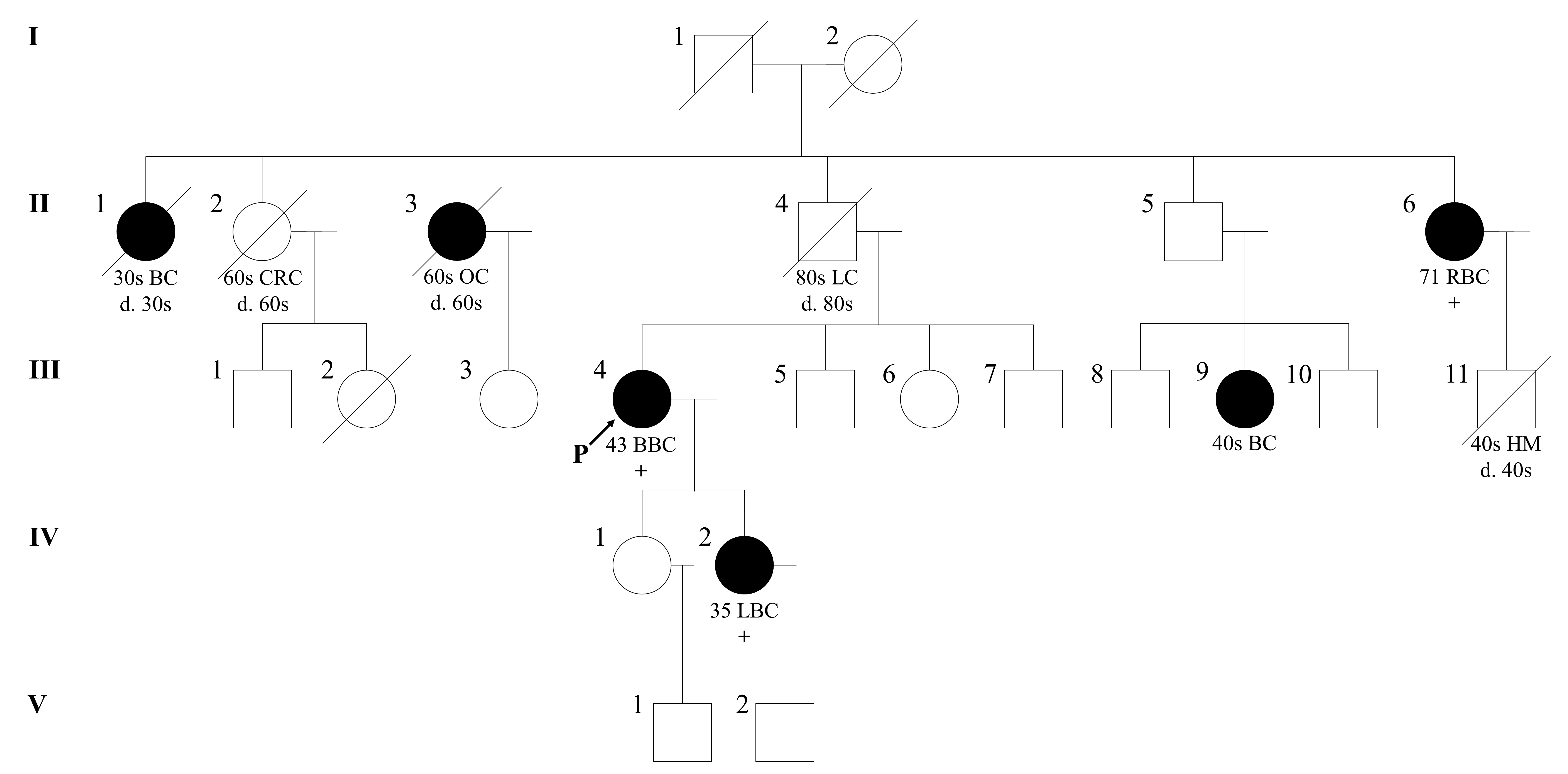


**Supplementary Figure S1.** Pedigree of three patients with *BRCA2* c.9221T>C p.(Leu3074Pro). The proband (III:4), her daughter (IV:2), and her aunt (II:6) underwent *BRCA1* and *BRCA2* sequencing testing and c.9221T>C p.(Leu3074Pro) was identified. The proband’s father (II:4) was supposed to have the same variant although confirmative sequencing testing was not performed. The proband was diagnosed with right breast cancer at the age of 43 years, left breast cancer at 55 years, and recurred right breast cancer at 61 years. The results of individuals who underwent genetic testing for c.9221T>C p.(Leu3074Pro) are indicated using either “+” (variant detected) or “−” (variant not detected).

Abbreviations: BBC, bilateral breast cancer; BC, breast cancer; CRC, colorectal cancer; HM, hematologic malignancy; LBC, left breast cancer; LC, lung cancer; OC, ovarian cancer; RBC, right breast cancer.
